# The immunomodulatory effects of lactoferrin and its derived peptides on NF‐κB signaling pathway: A systematic review and meta‐analysis

**DOI:** 10.1002/iid3.972

**Published:** 2023-08-10

**Authors:** Hojjat Allah Yami, Mojtaba Tahmoorespur, Ali Javadmanesh, Abbas Tazarghi, Mohammad Hadi Sekhavati

**Affiliations:** ^1^ Department of Animal Science, Faculty of Agriculture Ferdowsi University of Mashhad Mashhad Iran; ^2^ Stem Cell Biology and Regenerative Medicine Research Group, Research Institute of Biotechnology Ferdowsi University of Mashhad Mashhad Iran; ^3^ Department of Microbiology, Faculty of Medicine Golestan University of Medical Sciences Gorgan Iran

**Keywords:** immunomodulatory, lactoferrin, meta‐analysis, NF‐κB pathway

## Abstract

**Background:**

Lactoferrin is a versatile protein with important modulatory functions in inflammation and immune response. This glycoprotein can bind and sequester iron and LPS, thereby intervening in certain signaling pathways and biological processes. In the present meta‐analysis, we aimed to pool experimental data regarding the immunomodulatory effects of lactoferrin and its derived peptides on the NF‐κB signaling pathway.

**Materials:**

We searched PubMed, Google Scholar, and Web of Science databases and obtained all related articles published before April 2022. Finally, 25 eligible studies were selected, and their reports were analyzed.

**Methods:**

We used Review Manager Version 5.2 to compute the standardized mean difference (SMD) and its 95% confidence interval. In addition, the source of heterogeneity was explored using meta‐regression and sensitivity analysis. The symmetry of the funnel plot and Egger's test were also used to evaluate publication bias utilizing Comprehensive Meta‐Analysis Version 2.

**Results:**

Comparing the group of cells and animals exposed to lipopolysaccharide alone with the group that received pretreatment with lactoferrin and its derivatives, we observed significant reductions in TNF‐α, IL‐1 beta, and IL‐6 levels by 8.73 pg/mL, 2.21 pg/mL, and 3.24 pg/mL, respectively, in the second group. Additionally, IKK‐β, p‐IκB, and NF‐κB (p65) levels were significantly lower by 7.37‐fold, 15.02‐fold, and 3.88‐fold, respectively, in various cells and tissues.

**Conclusion:**

Based on the results of this meta‐analysis, lactoferrin and its derived peptides can be considered potent prophylactic and therapeutic candidates against inflammation‐associated diseases by targeting the NF‐kB pathway.

## Graphical abstract

This study is a meta‐analysis with the aim of systematic review of reported data to clarify the Immunomodulatory effects of lactoferrin and its derived peptides on NF‐κB signaling pathway. This meta‐analysis discovered that lactoferrin and its derivative peptides may have role of the modulators and Anti‐Inflammatory of immune processes by inhibitory effect on the TNF‐α, IL‐1β, and IL‐6 cytokines in upstream and IKK‐β, p‐IκB, and NF‐κB‐p65 in downstream of the NF‐κB signaling pathway.

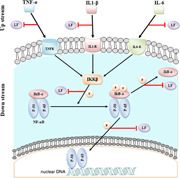

## 1 INTRODUCTION

Immunomodulators are natural or synthetic compounds that can inhibit and augment components of the immune system and alter immune responses.^1^ Some small proteins and peptides with immunomodulatory activity are produced in the human body.^2^ Lactoferrin (LF) is one of these glycoproteins abundantly present in the digestive fluids as well as in milk, tear, and saliva.^3^ It exhibits antimicrobial activity against various pathogens including viruses, parasites, fungi, and bacteria.^4^ Previous studies have shown that LF specifically binds to porins present in the outer membrane of gram‐negative bacteria,^5^ inducing the rapid release of lipopolysaccharide (LPS) and lysozyme, which results in osmotic shock and increases the susceptibility of bacteria to other antibacterial agents.^6^ LF also interacts with antigen‐presenting cells (e.g., macrophages, dendritic cells, and B‐cells) and bridges innate and adaptive immune functions to modulate T‐ and B‐cell responses.^7^ In addition, recent studies have reported that LF may even prevent interactions between viruses and some cellular receptors, such as heparan sulfate proteoglycans (HSPGs) and probably angiotensin‐converting enzyme 2 (ACE2). This suggests that LF might be a promising therapeutic supplement against COVID‐19.^8^, ^9^ Moreover, LF exerts an anti‐inflammatory effect by inducing immune cells to produce certain cytokines, such as interleukin (IL) and tumor necrosis factor (TNF).^10^ The mechanism by which LF activates intracellular signaling pathways to prompt the production of these cytokines is yet to be elucidated from a molecular perspective.^11^ Cytokines are chemical messengers that play a critical role in the modulation of the immune system by repairing damaged tissues, turning off the immune response, and regulating the cellular response against microorganisms and infectious and noninfectious tissue injuries. These glycoproteins are classified as regulatory, pro‐inflammatory, and anti‐inflammatory cytokines based on their influence.^12^

Nuclear factor‐kappa B (NF‐κB) transcription factor is involved in the regulation of several processes, namely apoptosis, cell growth, and immune responses.^13^ The NF‐κB signaling pathway bears a key function in regulating inflammation and inflammation‐related bone destruction.^14^, ^15^ IκB is an inhibitory protein that interacts with cytoplasmic NF‐κB in unstimulated cells. Pro‐inflammatory stimuli, such as LPS, and pro‐inflammatory cytokines, such as TNF‐α, IL‐6, and IL‐1β, cause phosphorylation and ubiquitination of IκB, which consequently leads to its proteasomal degradation.^16^ IκB kinase (IKK) complex with three subunits, including IKKα, IKKβ, and IKK/NF‐κB essential modulator (NEMO), significantly contributes to IκB phosphorylation.^17^

To the best of our knowledge, no previous systematic review and meta‐analysis of experimental data has examined the immunomodulatory effects of LF and its derived peptides on the NF‐κB signaling pathway. Therefore, in this study, we aimed to compile and consolidate experimentally reported data on LF exposure times and doses in different animals and cell lines to provide a theoretical basis for the potential application of this compound and its derived peptides in prophylactic and therapeutic interventions.

## 2 MATERIALS AND METHODS

### 2.1 Searching strategies

This meta‐analysis was performed according to the Preferred Reporting Items for Systematic Reviews and Meta‐Analyses (PRISMA). PubMed, Google Scholar, and Web of Science databases were searched for relevant articles published before April 2022. The search terms included “Lactoferrin” AND “Nuclear transcription factor kappa B” OR “NF‐Kappa B” OR “NF‐κB” OR “Inhibitor of kappa B” OR “IκB” OR “Inhibitor of kappa B kinase” OR “IKK.”

### 2.2 Inclusion and exclusion criteria

We were aiming to evaluate the immunomodulatory effects of LF and its derived peptides on LPS‐induced NF‐κB signaling pathway activation based on the pooled experimental meta‐analyses related to our subject of matter. Eligible articles were selected based on the following criteria: all animals and cell lines, no gender restrictions, and content published in English only. Review articles, unrelated studies, those with a lack of appropriate controls, incomplete information, and overlapping studies were excluded (Table 1). All selected in vitro and in vivo studies had been conducted based on the following methodology. First, the cells or animals of interest were divided into two groups called “control group” and “experimental group.” In the first phase of the experiments, the control group received no treatment, whereas the experimental group was treated with different types of LF and its derived peptides, which varied from one study to another. In the second phase, both groups were exposed to 1 μg/mL LPS. Finally, the protein levels of TNF‐α, IL‐1β, and IL‐6 were detected using ELISA kits, and real‐time qPCR analysis was performed to determine the mRNA levels of IKK‐β, p‐IκB, and NF‐κB (p65). Of note, there were studies in which different LF exposure times and doses had been applied. These studies were classified as follows: (a) ≤24 h versus (b) >24 h in terms of exposure time, and (c) ≤100 μg/mL (low dose) versus (d) >100 μg/mL (high dose) in terms of exposure dose. In these studies, the longest LF exposure time and highest LF exposure dose were selected.

**Table 1:** Main characteristics of the studies used in this meta‐analysis.

Authors	Year	*n*	Cells or tissue	Type of lactoferrin	Type of LPS	Testing method	References
Crouch	1992	7	MNC	Human lactoferrin	*Escherichia coli Olll:B4*	ELISA	[20]
Baltzer	1996	3	THP‐1	Bovine lactoferricin	*E. coli O127*	ELISA	[21]
Kruzel	2002	6	CF‐1 mice	Human lactoferrin	*E. coli Olll:B4*	ELISA	[22]
Berlutti	2006	3	Caco‐2	Bovine lactoferrin	*E. coli HB101*	ELISA	[23]
Li	2009	4	HC 11	Mouse lactoferrin	*E. coli Olll:B4*	qPCR	[24]
Ando	2010	3	THP‐1	Human lactoferrin	*E. coli Olll:B4*	ELISA/qPCR	[11]
Valenti	2011	3	IB3‐1	Bovine lactoferrin	*E. coli 055:B5*	ELISA	[25]
Inubushi	2012	3	OBs osteoblasts	Bovine lactoferrin	*E. coli Olll:B4*	ELISA	[26]
Inubushi	2012	3	ST2	Bovine lactoferrin	*E. coli Olll:B4*	qPCR	[26]
Cutone	2014	3	THP‐1	Bovine lactoferrin	*E. coli Olll:B4*	ELISA	[27]
Frioni	2014	3	CFBE	Bovine lactoferrin	*E. coli AIEC*	ELISA	[28]
Frioni	2014	3	Caco‐2	Bovine lactoferrin	*E. coli AIEC*	ELISA	[28]
Majka	2016	3	hMDM	Bovine lactoferrin	*E. coli Olll:B4*	ELISA	[29]
Rasheed	2016	3	Human chondrocytes	Camel lactoferrin	NR	qPCR	[30]
Mantuano	2016	6	BMDMs	Human lactoferrin	*E. coli Olll:B4*	qPCR	[31]
Zemankova	2016	6	HEK	Human lactoferrin	*E. coli Olll:B4*	qPCR	[32]
Zong	2016	3	IPEC‐1	Bovine lactoferricin	*E. coli Olll:B4*	qPCR	[33]
Hu	2017	3	PBMC	Human lactoferrin	*E. coli Olll:B4*	ELISA	[34]
Daniela	2017	3	THP–1	Bovine lactoferrin	*E. coli Olll:B4*	ELISA	[35]
Mohamed	2018	30	diabetic patients	Human lactoferrin	*E. coli Olll:B4*	ELISA/qPCR	[36]
Song	2019	12	RAW 264.7	Bovine lactoferricin	*E. coli O55:B5*	qPCR	[37]
Lutaty	2020	3	U937‐macrophages	Human lactoferricin	*E. coli 055:B5*	ELISA	[38]
Zhao	2020	3	BMDM	Human lactoferrin	*E. coli Olll:B4*	ELISA/qPCR	[39]
Yang	2020	3	PC‐12	Human lactoferrin	NR	qPCR	[40]
Li	2020	3	PC‐12	Human lactoferrin	NR	qPCR	[41]
Wright	2020	3	THP‐1	Human lactoferrin	*E. coli K12*	ELISA	[42]
Nemati	2021	3	RAW 264.7	Human lactoferrin	*E. coli 0127, B8*	ELISA/qPCR	[43]

Abbreviations: ELISA, enzyme linked immunosorbent assay; LPS, lipopolysaccharides; *n*, number of experiments; PCR; NR, not reported; qRT‐PCR, quantitative real‐time.

### 2.3 Data extraction and quality assessment

Basic information for the experimental and control groups (number of experimental cells or animal groups, mean and standard deviation [SD]), subject characteristics (cell and tissue sources, LF dose, and exposure time), publication characteristics (first author, publication date, title of the study, and journal title), sources of index estimates, and outcome measures were among the information extracted from eligible studies. Then, two review authors independently assessed the risk of bias in each of these studies according to the PRISMA recommendations.

### 2.4 Statistical analysis

Comprehensive Meta‐Analysis Version 2 and Review Manager Version 5.2 (The Nordic Cochrane) software programs were used to analyze 25 articles. Cochrane Q statistics and Higgins *I* squared statistical analyses (*I* ^2^ = [(Q−*df*)/Q] × 100%) were performed to assess research heterogeneity.^18^ The random‐effects model was used to calculate significant heterogeneity in *I* ^2^ values greater than 50% and a *p* < 0.05. To examine the sources of heterogeneity, we performed meta‐regression and subgroup analyses. A funnel plot was constructed, and Egger's linear regression test was performed to provide a more accurate assessment of publication bias.^19^ Sensitivity analysis was also carried out to test the stability and reliability of the results.

## 3 RESULTS

### 3.1 Study selection

**Figure 1:**
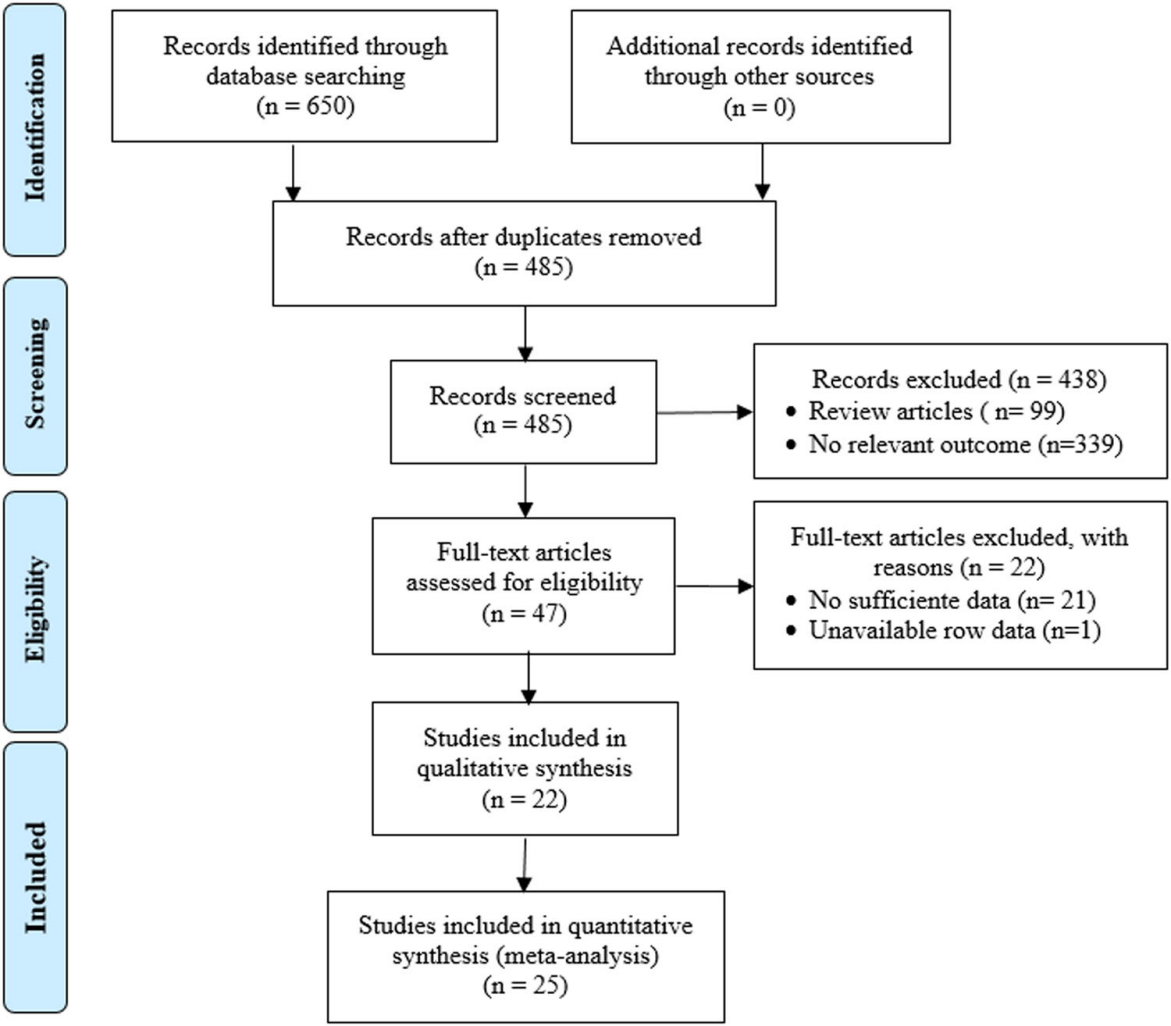
Flowchart of the criteria for study selection.

A total of 650 articles were screened from Google Scholar, Web of Science, and PubMed databases. In total, 165 duplicate publications were eliminated from the analysis, leaving 485 potentially relevant articles. After a second round of screening, 99 review articles, 339 articles with no relevant outcome, 21 articles with insufficient data, and one article with unavailable raw data were excluded. Finally, 25 studies were included in this meta‐analysis (Figure 1).

### 3.2 Study characteristics

From the 25 included articles, 23 cases were known to belong to group (a) (≤24 h, *n* = 23), and 2 cases were placed in group (b) (>24 h, *n* = 2) concerning the LF exposure time. Also, 19 studies were placed in group (c) low doses (≤100 μg/mL, *n* = 19), while six studies were included in group (d) high doses (>100 μg/mL, *n* = 6) regarding the LF exposure dose.

### 3.3 Upstream effects of lactoferrin

#### 3.3.1 TNF‐α cytokine

**Figure 2:**
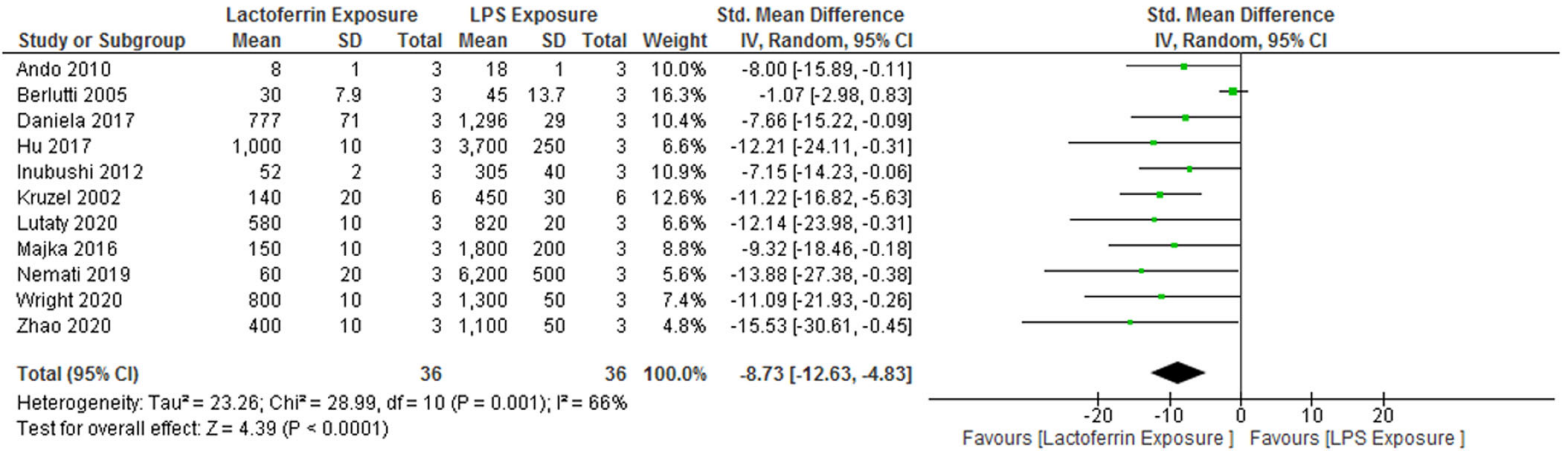
Forest plot for the comparison of TNF‐α cytokine levels between the experimental group and control group. CI, confidence interval; IV, independent variable.

Among the 25 selected studies, 11 cases had evaluated TNF‐α cytokine levels. The results showed significant heterogeneity (*p* = .001; *I* ^2^ = 66%), and the levels of TNF‐α cytokine (8.73 pg/mL) were lower in the lactoferrin‐exposed group than in the LPS‐exposed group (95% CI, −12.63 to −4.43; *Z* = 4.39; *p* < .0001) (Figure 2).

#### 3.3.2 IL‐1β cytokine

**Figure 3:**
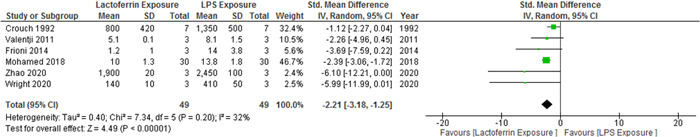
Forest plot for the comparison of IL‐1β cytokine levels between the experimental group and control group. CI, confidence interval; IV, independent variable.

Among the 25 selected studies, IL‐1β cytokine levels had been evaluated in six cases. The results revealed no significant heterogeneity (*p* = .2; *I* ^2^ = 32%), and the levels of IL‐1β cytokine (2.21 pg/mL) were lower in the lactoferrin‐exposed group than in the LPS‐exposed group (95% CI, −3.18 to −1.25; *Z* = 4.49; *p* < .00001) (Figure 3).

#### 3.3.3 IL‐6 cytokine

**Figure 4:**
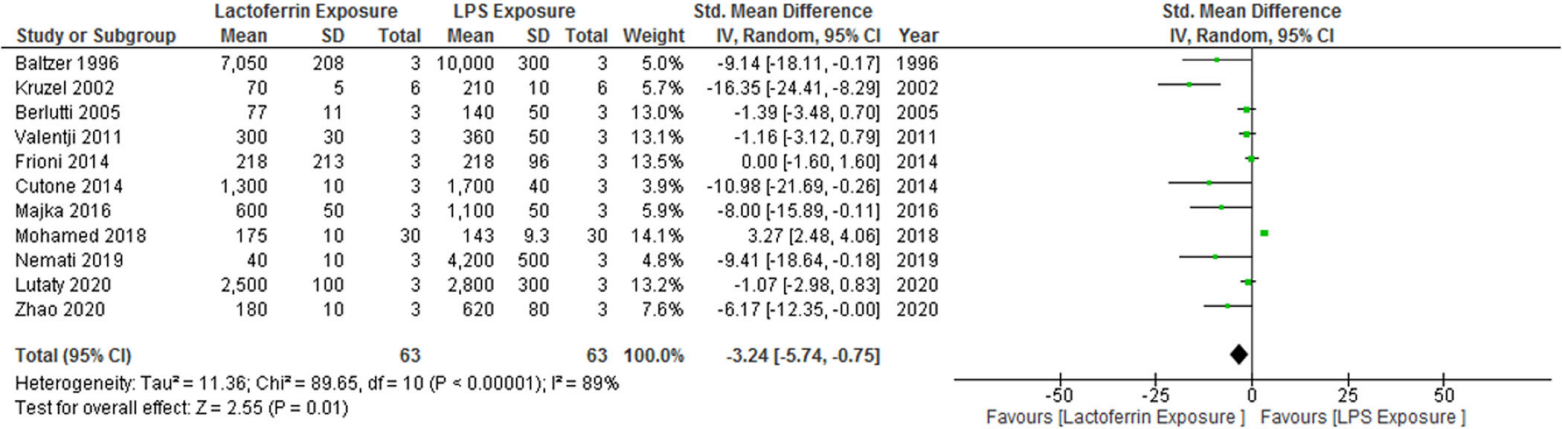
Forest plot for the comparison of IL‐6 cytokine levels between the experimental group and control group. CI, confidence interval; IV, independent variable.

Among the 25 selected studies, 11 cases had evaluated IL‐6 cytokine levels. The results showed significant heterogeneity (*p* < .00001; *I* ^2^ = 89%), and the levels of IL‐6 cytokine (3.24 pg/mL) were lower in the lactoferrin‐exposed group than in the LPS‐exposed group (95% CI, −5.74 to −0.75; *Z* = 2.55; *p* = .01) (Figure 4).

### 3.4 Downstream effects of lactoferrin

#### 3.4.1 IKK‐β

**Figure 5:**
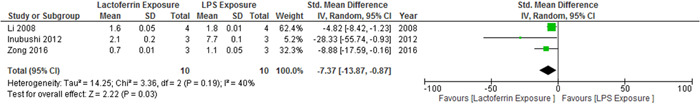
Forest plot for the comparison of IKK‐β levels between the experimental group and control group. CI, confidence interval; IV, independent variable.

Among the 25 selected studies, IKK‐β levels had been evaluated in three cases. The results showed no significant heterogeneity (*p* = .19; *I* ^2^ = 40%), and IKK‐β levels (7.37‐fold) were lower in the lactoferrin‐exposed group than in the LPS‐exposed group (95% CI, −13.87 to −0.871; *Z* = 2.22; *p* = .03 (Figure 5).

#### 3.4.2 p‐IκB α

**Figure 6:**
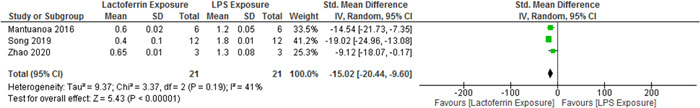
Forest plot for the comparison of p‐IκBα levels between the experimental group and control group. CI, confidence interval; IV, independent variable.

Among the 25 selected studies, three cases had evaluated p‐IκBα levels. The results showed no significant heterogeneity (*p* = .19; *I* ^2^ = 41%), and p‐IκBα levels (15.02‐fold) were lower in the lactoferrin‐exposed group than in the LPS‐exposed group (95% CI, −20.44 to −9.6; *Z* = 5.43; *p* < .00001) (Figure 6).

#### 3.4.3 NF‐kB (P65)

**Figure 7:**
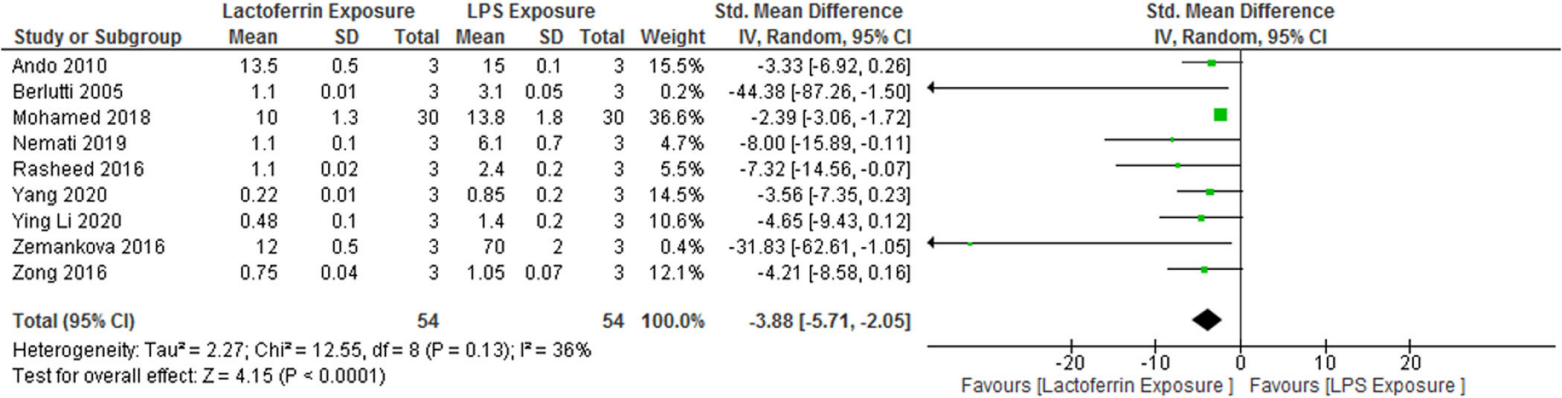
Forest plot for the comparison of NF‐κB (p65) levels between the experimental group and control group. CI, confidence interval; IV, independent variable.

Among the 25 selected studies, NF‐κB (p65) levels had been evaluated in nine cases. The results revealed no significant heterogeneity (*p* = .65; *I* ^2^ = 36%), and the levels of NF‐κB (p65) (3.88‐fold) were lower in the lactoferrin‐exposed group than in the LPS‐exposed group (95% CI, −5.71 to −2.05; *Z* = 4.15; *p* < .0001) (Figure 7).

### 3.5 Subgroup analyses

#### 3.5.1 Grouped by time

Our meta‐analysis demonstrated that LF exposure for ≤24 h reduced TNF‐α (*p* < .00001), IL‐6 (*p* = .0003), and IκBα phosphorylation (*p* < .00001), and induced weak inhibition of IKK‐β (*p* = .03) expression, whereas LF exposure for >24 h reduced IL‐1β (*p* < .00001) and suppressed NF‐κB (p65) activity (*p* < .00001).

#### 3.5.2 Grouped by dose

According to our findings, subgroup analysis showed that low LF dose (≤100 μg/mL in vitro or ≤100 mg/kg in vivo) inhibited IκBα phosphorylation (*p* < .00001), while high LF dose (>100 μg/mL in vitro or >100 mg/kg in vivo) decreased protein levels of TNF‐α (*p* < .0001), IL‐1β (*p* < .00001), and NF‐κB (p65) activity (*p* < .00001) (Table 2).

**Table 2:** Subgroup meta‐analysis of immunomodulatory effects of lactoferrin and its derived peptides on NF‐κB pathway.

Author	Year	lactoferrin	Experimental model (exposure dosage/time)	Inflammatory mediator investigated	Major findings
Crouch	1992	Human lactoferrin	The LF model group was exposed to 100 μg/mL hLf and the control group was exposed to 1 μg/mL LPS for 24 h	Pro‐inflammatory cytokine levels	IL‐1β (↓)
Baltzer	1996	Bovine lactoferricin	The LF model group was exposed to 50 μg/mL bLfcin peptide and the control group was exposed to 0.1 μg/mL LPS for 24 h	Pro‐inflammatory cytokine levels	IL‐6 (↓)
Kruzel	2002	Human lactoferrin	The LF model group was injected to 5 mg hLf and the control group was injected to 3·75 × 105 (EU/mouse) LPS for 18 h postinduction	Pro‐inflammatory cytokine levels	TNF‐α (↓) IL‐6 (↓)
Berlutti	2006	Bovine lactoferrin	The LF model group was exposed to 100 μg/mL bLf and the control group was exposed to 1 μg/mL LPS for 4 h	Pro‐inflammatory cytokine levels	TNF‐α (↓) IL‐6 (↓)
Li	2009	Mouse lactoferrin	The LF model group was exposed to 0.2 μg/mL mLF and the control group was exposed to 0.1 μg/mL LPS for 3 h	mRNA levels of IKK‐β	IKK‐β (↓)
Ando	2010	Human lactoferrin	The LF model group was exposed to 500 μg/mL hLf and the control group was exposed to 75 EU/mL LPS for 24 h	Pro‐inflammatory cytokine levels	TNF‐α (↓) NFkB(P65)(↓)
Valenti	2011	Bovine lactoferrin	The LF model group was exposed to 100 μg/mL bLf and the control group was exposed to 0.1 μg/mL LPS for 3 h	Pro‐inflammatory cytokine levels	IL‐1β (↓) IL‐6 (↓)
Inubushi	2012	Bovine lactoferrin	The LF model group was exposed to 100 μg/mL bLf and the control group was exposed to 0.1 μg/mL LPS for 48 h	Pro‐inflammatory cytokine levels	TNF‐α (↓)
Inubushi	2012	Bovine lactoferrin	The LF model group was exposed to 100 μg/mL bLf and the control group was exposed to 0.1 μg/mL LPS for 4 h	mRNA levels of IKK‐β	IKK‐β (↓)
Cutone	2014	Bovine lactoferrin	The LF model group was exposed to 100 μg/mL bLf and the control group was exposed to 1 μg/mL LPS for 24 h	Pro‐inflammatory cytokine levels	IL‐6 (↓)
Frioni	2014	Bovine lactoferrin	The LF model group was exposed to 100 μg/mL bLf and the control group was exposed to 1 μg/mL LPS for 24 h	Pro‐inflammatory cytokine levels	IL‐1β (↓) IL‐6 (↓)
Majka	2016	Bovine lactoferrin	The LF model group was exposed to 5000 μg/mL bLf and the control group was exposed to 0.1 μg/mL LPS for 24 h	Pro‐inflammatory cytokine levels	TNF‐α (↓) IL‐6 (↓)
Rasheed	2016	Bovine lactoferrin	The LF model group was exposed to 75 μg/mL bLf and the control group was exposed to nuclear extract for 2 h	NF‐kB(P65) activity	NFkB(P65)(↓)
Mantuano	2016	Camel lactoferrin	The LF model group was exposed to 0.15 μg/ml CLf and the control group was exposed to 0.1 μg/mL LPS for 8 h	Phosphorylation of IκB‐α	p‐IκB α (↓)
Zemankova	2016	Human lactoferrin	The LF model group was exposed to 100 μg/mL hLf and the control group was exposed to 1 μg/mL LPS for 6 h	NF‐kB(P65) activity	NFkB(P65)(↓)
Zong	2016	Human lactoferrin	The LF model group was exposed to 25 μg/mL LFP‐20 peptite and the control group was exposed to 1 μg/mL LPS for 24 h	mRNA levels of IKK‐β, phosphorylation of IκB‐α, NF‐kB(P65) activity	IKK‐β (↓) p‐IκB α (↓) NFkB(P65)(↓)
Hu	2017	Bovine lactoferricin	The LF model group was exposed to 15 μg/mL bLfcin and the control group was exposed to 3 μg/mL LPS for 24 h	Pro‐inflammatory cytokine levels	TNF‐α (↓)
Daniela	2017	Human lactoferrin	The LF model group was exposed to 40 μg/mL hLf and the control group was exposed to 10 μg/mL LPS for 8 h	Pro‐inflammatory cytokine levels	TNF‐α (↓)
Mohamed	2018	Bovine lactoferrin	The LF model group was treated to oral LF capsules (250 mg/day) and the control group was standard therapy without LF for 3 months	Pro‐inflammatory cytokine levels, NF‐κB (P65) activity	IL‐1β (↓) IL‐6 (↑) NFkB(P65)(↓)
Song	2019	Human lactoferrin	The LF model group was exposed to 10 μg/mL LFCA peptite and the control group was exposed to 1 μg/mL LPS for12h	Phosphorylation of IκB‐α	p‐IκB α (↓)
Lutaty	2020	Bovine lactoferricin	The LF model group was exposed to 10 μm FKD peptite and the control group was exposed to 0.1 μg/mL LPS for 24 h	Pro‐inflammatory cytokine levels	TNF‐α (↓) IL‐6 (↓)
Zhao	2020	Human lactoferricin	The LF model group was exposed to 50 μg/mL hLfcin and the control group was exposed to 0.1 μg/mL LPS for 1 h	Pro‐inflammatory cytokine levels, phosphorylation of IκB‐α	TNF‐α (↓) IL‐1β (↓) IL‐6 (↓) p‐IκB α (↓)
Yang	2020	Human lactoferrin	The LF model group was exposed to 100 mg/kg hLf and the control group was exposed to 1 mg/mL OGD for 4 h	NF‐κB (P65) activity	NFkB(P65)(↓)
Li	2020	Human lactoferrin	The LF model group was exposed to 100 mg/kg hLf and the control group was exposed to 1 mg/mL OGD for 48 h	NF‐κB (P65) activity	NFkB(P65)(↓)
Wright	2020	Human lactoferrin	The LF model group was exposed to 100 μg/mL hLf and the control group was exposed to 0.05 μg/mL LPS for 6 h	Pro‐inflammatory cytokine levels	TNF‐α (↓) IL‐1β (↓)
Nemati	2021	Human lactoferrin	The LF model group was exposed to 500 μg/mL hLf and the control group was exposed to 1 μg/mL LPS for 24 h	Pro‐inflammatory cytokine levels, NF‐κB (P65) activity	TNF‐α (↓) IL‐6 (↓) NFkB(P65)(↓)

Abbreviations: bLf, bovine lactoferrin; bLfcin, bovine lactoferricin; cLf, camel lactoferrin; hLf, human lactoferrin; hLfcin, human lactoferricin; mLf, mouse lactoferrin.

#### 3.5.3 Sensitivity analysis and meta‐regression

Sensitivity analysis was performed to test the stability of the results. No individual study was found to influence the combined results, indicating that the results of these studies were stable. As the heterogeneity between LF exposure and TNF‐α and IL‐6 levels was striking, meta‐regression was used to explore the source of heterogeneity. The results showed that the influence of LF exposure time and dose on TNF‐α and IL‐6 cytokine levels was significant (*p* < .05).

#### 3.5.4 Publication bias

**Figure 8:**
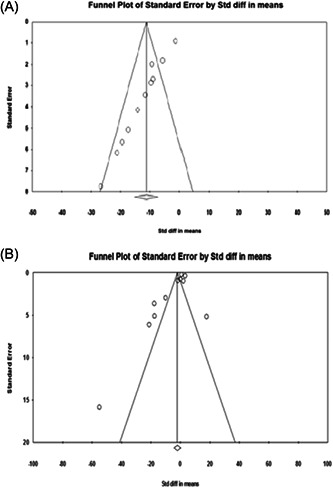
Funnel plot of the association between lactoferrin exposure and TNF‐α levels (A) and IL‐6 levels (B). No evidence of publication bias was found. SE, standard error; SMD, standard mean difference.

Egger's test was performed and a funnel plot was generated to check for publication bias. Egger's linear regression analysis revealed no statistically significant bias for TNF‐α (*p* = .059) and IL‐6 levels (*p* = .073). Moreover, the funnel plot did not show any obvious asymmetry (Figure 8).

## 4 DISCUSSION

LF is a glycoprotein containing 700–720 amino acids, with high homology between species. It belongs to the family of transferrins, mostly present in certain body fluids, such as bile, saliva, tears, gastrointestinal secretions, and especially in milk. The concentration of LF varies from 1 mg/mL in mature milk to 7 mg/mL in colostrum. It is secreted in the form of apolipoproteins from epithelial cells and plays a crucial role in host defense mechanisms. LF binds and sequesters iron. It also interacts with lipoproteins, apolipoproteins, proteoglycans, lymphocytes, enterocytes, and nucleolins.^3^ As an integral part of the innate immune system, LF is a well‐known immunomodulator of leukocyte populations that performs its regulatory function by inhibiting several cytokines.^20^, ^44^

The ability of LF to bind and neutralize LPS through its N‐terminal domain limits the interaction of this pathogen‐associated molecular pattern (PAMP) with cellular or soluble receptors and reduces the intensity of the LPS‐mediated inflammatory response of phagocytic cells. LPS is the main component of the outer membrane of gram‐negative bacteria and is known to initiate inflammation.^45^ LPS causes rapid changes in the expression of cell surface receptors in human monocytes. These interactions result in cell capture and the induction of biological responses, details of which have remained controversial. LF through its lactoferricin domain neutralizes LPS and downregulates pro‐inflammatory cytokines. However, it has been suggested that the complex of LF with LPS (LF‐LPS) suppresses the anti‐inflammatory effects of LF when it is added before LPS. In addition, the amount of intracellular LF directly correlates with its inhibitory effects. This explains why the maximal inhibitory impact of LF depends on the time at which it is added in relation to LPS, and different LF doses must be evaluated to identify the most effective one against LPS stimulation.^46^, ^47^

Zemankova and colleagues reported that the administration of LF to LPS‐exposed native mice 1 h before LPS administration causes a decline in serum TNF‐α and IL‐6 levels, while exposure to LF 18 h before administration of LPS has a preventive effect on TNF‐α production but not on IL‐6.^32^ The ability of LF to bind to LPS and reduce TNF‐α production in endotoxin shock, along with its direct bacteriostatic and protective nature in the gut structure, may make it a promising agent for clinical application as a postoperative treatment.^48^, ^49^, ^50^ Sorimachi et al.^10^ unanimously confirmed that LF supplementation before or after the addition of LPS has an inhibitory effect on TNF‐α and IL‐6 secretion. Moreover, Yen and colleagues reported that after LF treatment, the levels of TNF‐α, IL‐1ß, and IL‐6 decreased, and the protein level of IκB markedly increased in transgenic mice with hyperoxia‐induced lung injury.^51^

Kruzel and colleagues asserted that when LF and LPS are administered concurrently, the activity of anti‐ and pro‐inflammatory mediators is significantly suppressed, which may be due to the generalized deactivation of monocytes and macrophages. In this co‐treatment protocol, 2 h after the addition of LF‐LPS, the levels of TNF‐α, IL‐6, and IL‐10 decreased by 82%, 43%, and 47%, respectively.^22^

Similar to human LF (hLF), bovine LF (bLF) has also been observed to exert modulatory effects (LPS‐mediated and basal) on the release of pro‐inflammatory cytokines such as TNF‐α, which is an essential mediator in both infectious and immune responses.^21^, ^52^ The internalization of bLF is primarily mediated by the endocytic receptor LRP1, a member of the low‐density lipoprotein receptor family and ubiquitously expressed type 1 membrane protein.^53^ Inubushi and colleagues demonstrated that 4 h after treatment with bLF, the internalized bLF bound to endogenous TNF receptor‐associated factor 6 (TRAF6) and interfered with its auto‐ubiquitination and activation of the NF‐κB signaling pathway. NF‐κB activation involves two major mechanisms that respond to diverse stimuli including pro‐inflammatory cytokines. Inubushi et al. found that IKK phosphorylation and IκB degradation were effectively blocked by bLF pretreatment, similar to the blockage of c‐Jun N‐terminal kinase (JNK) and p38 phosphorylation.^26^ Liu and colleagues observed that LF suppresses the activation of NF‐κB (p65) by downregulating the expression levels of macrophage‐associated chemokines Ccl2 and Ccl5 and that the highly activated p65 signaling pathway aggravates LPS‐induced acute inflammation in mice.^54^ Wei and colleagues expressed that LF can improve intestinal injury and survival time in irradiated mice by lowering NF‐κB, TNF‐α, and IL‐6 levels.^55^ Valenti and colleagues showed that bLF differentially regulated inflammatory responses in a two‐epithelial model. In particular, 4 h of exposure to bLF did not regulate the inflammatory responses in either uninfected or *Burkholderia cenocepacia*‐infected cells in a bronchial epithelium model, whereas in the infected cystic fibrosis epithelial version, the levels of pro‐inflammatory cytokines were significantly reduced after 24 h of exposure to bLF.^25^ Na and colleagues demonstrated that LF can regulate the DNA‐binding activity of NF‐κB, and pretreated RAW 264.7 cells experience LPS tolerance accompanied by a decline in TNF‐α, IL‐1β, and IL‐6 levels.^56^ Mohamed et al. also revealed that LF inhibits Toll‐like receptor 4 (TLR4) in type 2 diabetes by promoting the activation of peroxisome proliferator‐activated receptor gamma (PPAR‐γ) and sirtuin 1 (SIRT‐1) proteins in the downstream NF‐κB signaling pathway.^36^

It also has been revealed that at low doses (e.g., 0.01–1 µg/mL), hLF inhibits IL‐6 secretion in LPS‐activated THP‐1 cells,^21^ whereas low doses of bLF (0.1–1.0 mg/mL) reduce TNF‐α cytokine secretion in LPS‐ativated THP‐1 and LPS‐ativated RAW 264.7 cells.^52^ Ando et al.^11^ showed that in the intestines of breast‐fed infants, hLF acts as an antagonist of LPS to inhibit NF‐κB activation and as an endogenous activator of TLR4 at low doses (25–500 µg/mL). In addition to LF, its peptide derivative, lactoferricin (LFcin), appears to cause similar outcomes. LFcin results from enzymatic cleavage of LF by pepsin in its N‐terminal domain. Drago‐Serrano and colleagues reported that LF and LFcin have nonspecific immunomodulatory roles in TLRs, pro‐ and anti‐inflammatory cytokines, and reactive oxygen species. Bovine lactoferricin (bLFcin) and human lactoferricin (hLFcin) are peptide fragments (residues 1–47 in hLF and 17–41 in bLF), consisting of two subunits linked by a disulfide bridge between cysteines. Despite the antigenic and chemical differences between bLF and hLF or hLFcin, both bLF and its fragments are recognized to exhibit even more potent suppressive effects on IL‐6 in human cells.^21^

**Figure 9:**
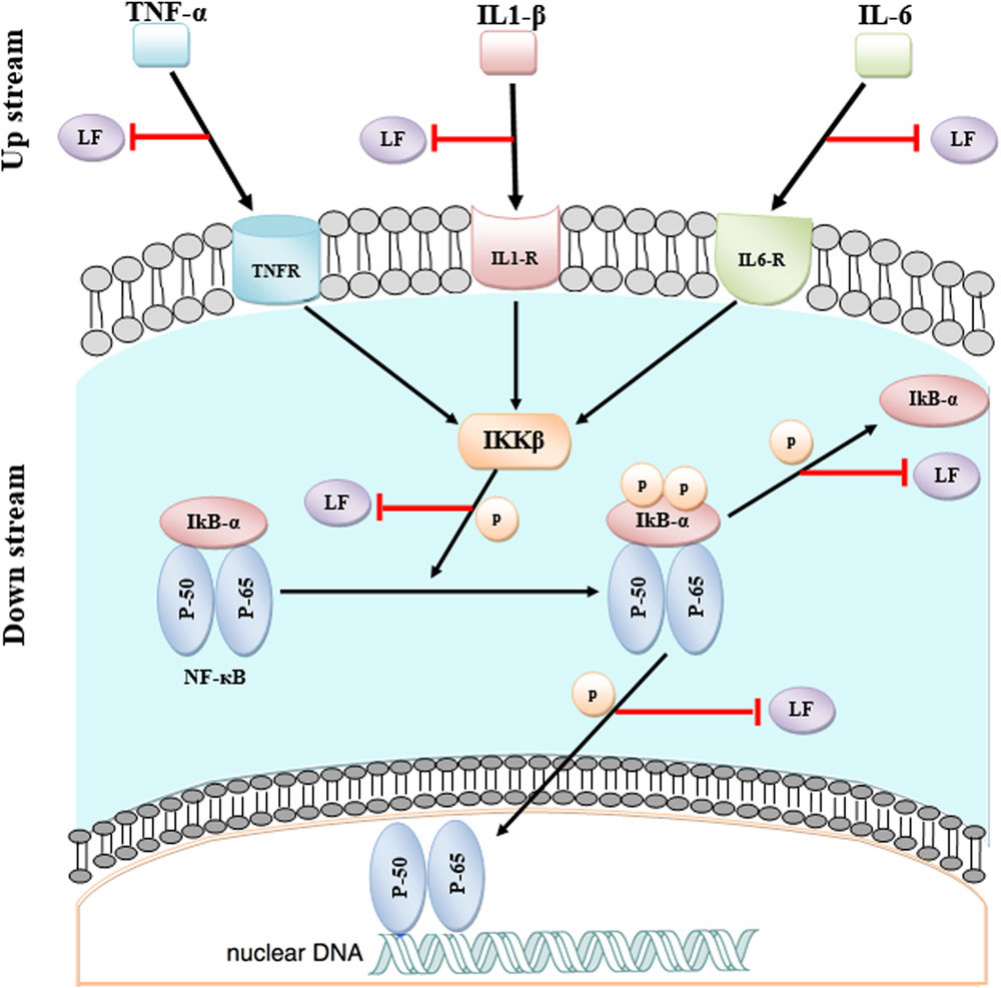
Inhibitory effects of lactoferrin on the upstream (IL‐6, TNF‐α, and IL‐1β cytokines) and downstream (IκBα, IKK‐β, and NF‐κB (p65) cytoplasmic) NF‐κB pathways.

Overall, the results of this meta‐analysis suggest that different types of LF and its derivatives, including hLF, bLF, hLFcin, and bLFcin, at different exposure times and doses probably exert a certain range of immunomodulatory and anti‐inflammatory effects on LPS‐induced cytokine production in various cells and tissues by reducing the levels of pro‐inflammatory cytokines (e.g., TNF‐α, IL‐1β, and IL‐6). These cytokines activate NF‐κB, which generates a positive feedback loop in the nucleus and promotes the transcription of NF‐κB‐dependent genes, including those responsible for the production of TNF‐α, IL‐1β, and IL‐6. Furthermore, we believe that the aforementioned types of LF also modulate the proteins involved in the downstream NF‐κB signaling pathway (e.g., IKK‐β, IκBα, and NF‐κB [p65]), indicating that the activity of all interacting elements associated with NF‐κB activation is dependent on the precise nature and intensity of both upstream and downstream regulatory signals. We observed that short LF exposure times may (a) reduce TNF‐α and IL‐6 cytokine levels, (b) decrease the phosphorylation of IκBα, and (c) induce weak inhibition of IKK‐β expression, whereas long LF exposure times may reduce IL‐1β cytokine levels and suppress NF‐κB (p65) activity (Figure 9). Moreover, our analyses indicated that low‐dose LF exposure inhibited the phosphorylation of IκBα, whereas high‐dose LF exposure reduced TNF‐α and IL‐1β levels as well as NF‐κB (p65) activity. These results are consistent with those of other studies that strengthen the reliability of our findings. This meta‐analysis had numerous obstacles that must be considered thoroughly. Differences in the effects of LF supplementation before and after the addition of LPS and an insufficient number of studies that did not allow subgroup analyses are important limitations that detrimentally affect the quality of the final results. It is also noteworthy to mention that pooling the related information and details of subgroup analyses was performed based on a random‐effects model, because heterogeneity is often a common limiting factor in most meta‐analysis studies.

## 5 CONCLUSIONS

The present meta‐analysis revealed that LF and its derived peptides might have modulatory and anti‐inflammatory roles in immune responses. LF and its derived peptides were observed to be capable of affecting the upstream and downstream NF‐κB pathways by reducing TNF‐α, IL‐6, and IL‐1β cytokine levels and suppressing the activity of IKK‐β, p‐IκB, and NF‐κB (p65), respectively. Based on our findings, LF and its derived peptides can probably be used in prophylactic and therapeutic approaches by targeting the NF‐kB pathway and inhibiting subsequent inflammation. Ultimately, despite the protective nature of LF and its derivatives, further in vivo and in vitro studies are warranted to explore the interactions between these peptides and inflammatory genes. We suggest that in silico molecular dynamics simulation studies can help unravel the intracellular mechanisms involved in the NF‐κB signaling pathway and its association with LF.

## AUTHOR CONTRIBUTIONS

Hojjat Allah Yami designed the study and developed the retrieval strategy. Hojjat Allah Yami and Mohammad Hadi Sekhavati screened the literature. Hojjat Allah Yami and Mohammad Hadi Sekhavati extracted the data from the literature. Hojjat Allah Yami, Mojtaba Tahmoorespur, and Mohammad Hadi Sekhavati conducted the meta‐analysis and wrote the manuscript. Abbas Tazarghi, Mojtaba Tahmoorespur, and Ali Javadmanesh proofread and revised the manuscript. All authors have read and approved the final revision.

## CONFLICT OF INTEREST STATEMENT

The authors declare no conflict of interest.

## Data Availability

The data that support the findings of this study are available from the corresponding author upon reasonable request.
